# Community acceptance of yeast interfering RNA larvicide technology for control of *Aedes* mosquitoes in Trinidad

**DOI:** 10.1371/journal.pone.0237675

**Published:** 2020-08-14

**Authors:** Akilah T. M. Stewart, Nikhella Winter, Jessica Igiede, Limb K. Hapairai, Lester D. James, Rachel Shui Feng, Azad Mohammed, David W. Severson, Molly Duman-Scheel

**Affiliations:** 1 Department of Life Sciences, The University of the West Indies at St. Augustine, St. Augustine, Trinidad and Tobago; 2 Eck Institute for Global Health, University of Notre Dame, Notre Dame, Indiana, United States of America; 3 Department of Biological Sciences, University of Notre Dame, Notre Dame, Indiana, United States of America; 4 Department of Medical and Molecular Genetics, Indiana University School of Medicine, South Bend, Indiana, United States of America; Universita degli Studi di Camerino, ITALY

## Abstract

RNA interference (RNAi), a technique used to investigate gene function in insects and other organisms, is attracting attention as a potential new technology for mosquito control. *Saccharomyces cerevisiae* (baker’s yeast) was recently engineered to produce interfering RNA molecules that silence genes required for mosquito survival, but which do not correspond to genes in humans or other non-target organisms. The resulting yeast pesticides, which facilitate cost-effective production and delivery of interfering RNA to mosquito larvae that eat the yeast, effectively kill mosquitoes in laboratory and semi-field trials. In preparation for field evaluation of larvicides in Trinidad, a Caribbean island with endemic diseases resulting from pathogens transmitted by *Aedes* mosquitoes, adult residents living in the prospective trial site communities of Curepe, St. Augustine, and Tamana were engaged. Open community forums and paper surveys were used to assess the potential acceptability, societal desirability, and sustainability of yeast interfering RNA larvicides. These assessments revealed that Trinidadians have good working knowledge of mosquitoes and mosquito-borne illnesses. A majority of the respondents practiced some method of larval mosquito control and agreed that they would use a new larvicide if it were proven to be safe and effective. During the community engagement forums, participants were educated about mosquito biology, mosquito-borne diseases, and the new yeast larvicides. When invited to provide feedback, engagement forum attendees were strongly supportive of the new technology, raised few concerns, and provided helpful advice regarding optimal larvicide formulations, insecticide application, operational approaches for using the larvicides, and pricing. The results of these studies suggest that the participants are supportive of the potential use of yeast interfering RNA larvicides in Trinidad and that the communities assessed in this investigation represent viable field sites.

## Introduction

Mosquito-borne illnesses such as dengue, Zika, chikungunya, and yellow fever result from infections with viruses spread primarily through the bites of infected female *Aedes* mosquitoes. Over one-third of the world’s population is at risk for contracting dengue, one of the most significant mosquito-borne diseases in the tropics and subtropics, which afflicts roughly 400 million people annually [1]. In 2019, over three million cases of dengue were recorded in the Americas, the largest number of cases recorded in the history of dengue in the region [2]. Trinidad and Tobago has an ongoing risk of dengue transmission, with the most recent outbreak occurring in 2014, at which time 5,157 probable dengue cases were recorded [3]. 575 suspected cases of chikungunya were recorded during the most recent Trinidad and Tobago outbreak in 2016 [3]. A Zika outbreak also occurred in Trinidad and Tobago in 2016, at which time 300 cases had been reported in pregnant women by epidemiology week (EW) 38 [3]. This was of major concern given that the virus can be transmitted from the mother to the fetus, which is at increased risk for developing severe congenital defects [4].

In Trinidad and Tobago, as in other arboviral disease endemic countries, vector control is the primary means of preventing mosquito-borne illnesses. *Aedes aegypti*, which is widely distributed throughout the Caribbean, is the primary arboviral vector mosquito in Trinidad and Tobago [5–7]. *Aedes albopictus*, a secondary arboviral vector, was identified in the Chaguaramas region of Trinidad in 2003 [8] and has since spread across the island. *A*. *aegypti* and *A*. *albopictus* mosquitoes lay eggs in water-filled containers located within or in close proximity to human dwellings [9]. Larviciding, the treatment of container breeding sites with microbial or chemical agents that kill larvae, is therefore a major component of integrated *Aedes* mosquito control and disease prevention programs in Trinidad and Tobago [10]. In addition to targeting adult mosquitoes with ultra-low volume malathion, the Insect Vector Control Division of the Trinidad and Tobago Ministry of Health (MoH) targets *Aedes* juveniles through larval source reduction campaigns and larvicide focal treatments with *Bacillus thuringiensis israelensis* (Bti) and Aquatain AMF^TM^ [11]. Unfortunately, due to increased insecticide resistance and escalating concerns for the negative effects of pesticides on non-target species, *Aedes* mosquitoes are becoming increasingly difficult to control in in Trinidad and Tobago [12–14] and throughout the world [9]. Identifying new strategies for controlling these mosquitoes is of vital importance.

Although it is attracting attention in agricultural pest biotechnology communities [15], the use of RNA interference (RNAi) is an innovative and still largely unexplored approach for mosquito control. The RNAi pathway is initiated by Dicer, which cleaves long pieces of double stranded.

RNA (dsRNA) into small interfering RNA (siRNA) molecules, which silence genes that are complementary in sequence. Recent high-throughput screens identified hundreds of interfering RNA larvicides that silence genes which are critical for mosquito larval survival [16, 17]. A subset of these interfering RNA larvicides recognize target sites that are conserved in multiple mosquito species, including *A*. *aegypti* and *A*. *albopictus*, but which are not found in non-target organisms. Broad use of these larvicides for mosquito control requires the identification of effective, affordable, and stakeholder-accepted means of introducing the RNAi larvicides to mosquito control programs worldwide. Evaluation of multiple interfering RNA delivery mechanisms in the laboratory resulted in the identification of baker’s yeast *(Saccharomyces cerevisiae)* as a promising RNA expression and delivery system [18].

The genetic tractability of *S*. *cerevisiae*, a genetic model organism, has facilitated generation of numerous yeast strains that express larvicidal interfering RNAs. Yeast is a strong odorant attractant to mosquito larvae, which readily consume larvicidal yeast. The larvicidal properties of the yeast strains are preserved upon heat inactivation [16, 17], an important discovery that would permit the use of dead microbes, rather than live genetically modified organisms, for mosquito control in the field. Moreover, interfering RNA can be propagated through yeast cultivation, which could make this intervention cost-effective. Fermentation processes are readily scaled from small laboratory-sized shake cultures to much larger production scale cultures. Yeasts have been cultivated worldwide for thousands of years, and this technology can be adapted to resource-limited countries with constrained infrastructures. Dried yeast can also be packaged and shipped, facilitating regional distribution of yeast pesticides. *S*. *cerevisiae*, which is non-toxic to humans, is sold as a dietary supplement and used globally in food and alcoholic beverage and food preparation. It is therefore hypothesized that interfering RNA could be delivered in dried yeast prepared in bulk and distributed in a ready-to-use tablet formulations, and that this technology would be broadly accepted by consumers [18]. In support of this, recent laboratory and semi-field investigations have confirmed the efficacy of dried yeast larvicidal tablets [19–21], and the potential for scaled production of commercial-ready formulations is presently being investigated [18]. This investigation evaluated potential stakeholder acceptance of this new intervention in Trinidad.

Stakeholder input is crucial for the ultimate acceptability and practical efficacy of novel interventions [22]. As described by Lavery et al. [22], effective community engagement in global health studies involves the early initiation of engagement activities. A consumer engagement approach that embodied pursuit of Responsible Research and Innovation (RRI) [23] was therefore developed. RRI is an interactive and transparent process by which societal actors and innovators become mutually responsive to each other while assessing the acceptability, societal desirability, and sustainability of the innovation process and its marketable products [23]. To this end, community engagement forums and paper surveys were used to assess stakeholders living in prospective field site communities in St. Augustine, Trinidad and the surrounding region. These assessment tools examined participants’ general knowledge of mosquitoes and mosquito control practices, queried participants regarding current mosquito control efforts in Trinidad, and tested the hypothesis that Trinidadian stakeholders would approve of yeast interfering RNA larvicide technology. Here, we present analysis of five community engagement events and results from paper surveys conducted in Trinidad throughout 2018. These studies revealed broad stakeholder acceptance of yeast interfering RNA technology in Trinidad.

## Methods

### Ethics statement

Stakeholder engagement studies (forum and paper surveys) were approved by the Indiana University (IU) Human Subjects Office (protocol 1608074907A008), the University of Notre Dame (ND) Office of Research Compliance (Protocol #17-07-3984), the Trinidad and Tobago Southwest Regional Health Authority Ethics Committee, and the University of the West Indies at St. Augustine, Trinidad and Tobago (UWI) Ethics Committee (Study CEC403/12/17).

### Community engagement forums

A total of five community engagement forums were held at prospective study sites in Trinidad, including two on the UWI campus, one in St. Augustine, one in Tamana, and one in Curepe. The locations, dates, and number of participants in attendance at each forum are summarized in Table 1. Guest attendance was incentivized through provision of refreshments during the events, which were advertised on posters, through electronic notices of events, using a car equipped with a loud speaker to announce the events (a typical means of advertising in Trinidad), and by recruiting passersby 30 minutes prior to the event. Attendees, who were required to be Trinidad residents age 18 or older, were provided a study information sheet (S1 File) prior to the start of the forum and were encouraged, though not required, to fill out demographic information sheets (S2 File). No individually identifying information was collected at the events.

**Table 1:** Summary of engagement forum events and attendance.

Engagement Forum	Location	Date	# of Attendees
UWI 1	UWI classroom, St. Augustine	3/15/18	16
UWI 2	UWI classroom, St. Augustine	4/5/18	35
Tamana	Tamana Presbyterian School	4/4/18	8
Curepe	Bushe Street Residential Home, Curepe	11/18/18	14
St. Augustine	St. Augustine South Community Ctr.	11/19/18	12

The locations of the engagement forums, dates, and number of attendees are noted.

Native Trinidadian members of the UWI research team moderated the events. At the beginning of each event, the moderator introduced herself and other members of the UWI, IU, and ND research teams who were in attendance. A short introduction to the project, including an overview of mosquitoes and their aquatic breeding sites, pathogens transmitted by *Aedes*, conventional control methods, and yeast interfering RNA larvicide technology, was provided. The moderator then led the participants through discussion of a series of scripted questions (S3 File) regarding the respondents’ feelings about the larvicides. Scientific experts from the IU and ND research teams then answered scientific questions that arose during the sessions. Audio recordings of each session were captured and used to generate transcripts that were prepared by native Trinidadian UWI staff members.

The transcripts were analyzed in detail. First, the participants’ remarks were coded using the following tags: 1) information-seeking questions, 2) positive comments, 3) negative comments, 4) neutral comments, and 5) advice regarding product design. All transcripts were coded independently by three members of the team. Codes generated by each individual were compared to check for differences, which were then reconciled by the coding team. Word count analyses of the combined responses from all five sessions were performed using TextAnalyzer [24], which uncovered repeated words and phrases, revealing further patterns in the transcript data. The results of these analyses, in addition to inspection of the transcripts by the coding team, were used as a basis to further code participant responses. Representative quotes that illustrate the overall sentiments found among each set of coded responses were selected by the research team.

### Paper surveys

Paper surveys were distributed to Trinidad residents 18 years of age and older and collected by the UWI staff members at several sites, including the UWI campus, the East-West Corridor (which includes the Tunapuna-Piarco, San Juan-Lavantille and Sangre Grande Regional Corporations), the Diego-Martin-Port of Spain (POS) region (Diego Martin and Port of Spain Regional Corporations), as well as the South (Penal-Debe, Princess Town and Siparia Regional Corporations) and Central (Chaguanas and Couva-Tabaquite-Talparo Regional Corporations) regions of Trinidad (Table 2). The surveys were conducted over a one year period from March 2018 through March 2019. The surveys were primarily administered to participants at public sites (i.e. shopping malls, UWI campus fairs, engagement forums) and occasionally at participants’ residences and immediately collected by the UWI staff. Individuals who participated in the engagement forums were invited to participate, and those that did completed the surveys prior to forum participation, ensuring that their participation in the forum did not skew their responses. Respondents were provided a study information sheet (S4 File) prior to completing the paper survey (S5 File), which consisted of ten Likert scale items designed to probe the general knowledge, current practices, and feelings of Trinidadian stakeholders about mosquitoes and mosquito control. The survey included an optional section that facilitated collection of demographic data, but no individually identifying information was collected with the surveys. If a participant requested assistance, staff members would read the questions to them and enter the participant’s responses; in these instances, the staff members did not advise the participant how to answer the question.

**Table 2:** Paper survey collection in Trinidad.

Location collected	Number Collected	% of Total
Central	117	23%
Diego-Martin-POS	85	17%
East-West Corridor	100	20%
South	39	8%
UWI	168	33%
Total	509	100%

The total numbers and percentages of the total surveys collected are noted for each location.

Survey responses were entered into an Excel spreadsheet that was compatible with import and subsequent data analysis with Qualtrics StatsIQ [25] software (Qualtrics, Provo, UT), which was used for data analysis. Likert scale responses to the survey questions were statistically evaluated using Qualtrics Stats iQ software. Statistical comparisons were conducted between responses to each variable (an individual paper survey question or demographic response) and all other variables (other paper survey questions) in the data set. Statistically significant results observed in these analyses are reported and discussed herein. The Stats iQ software recommends the most appropriate statistical test for each comparison, and the statistical comparisons reported herein adhered to these recommendations. The Stats iQ software alerts researchers when the size of the data set is too small for statistical comparisons to be reliable, and data flagged by such alerts are not reported here.

## Results and discussion

### Community engagement forums

#### Participant demographics

**Fig 1:**
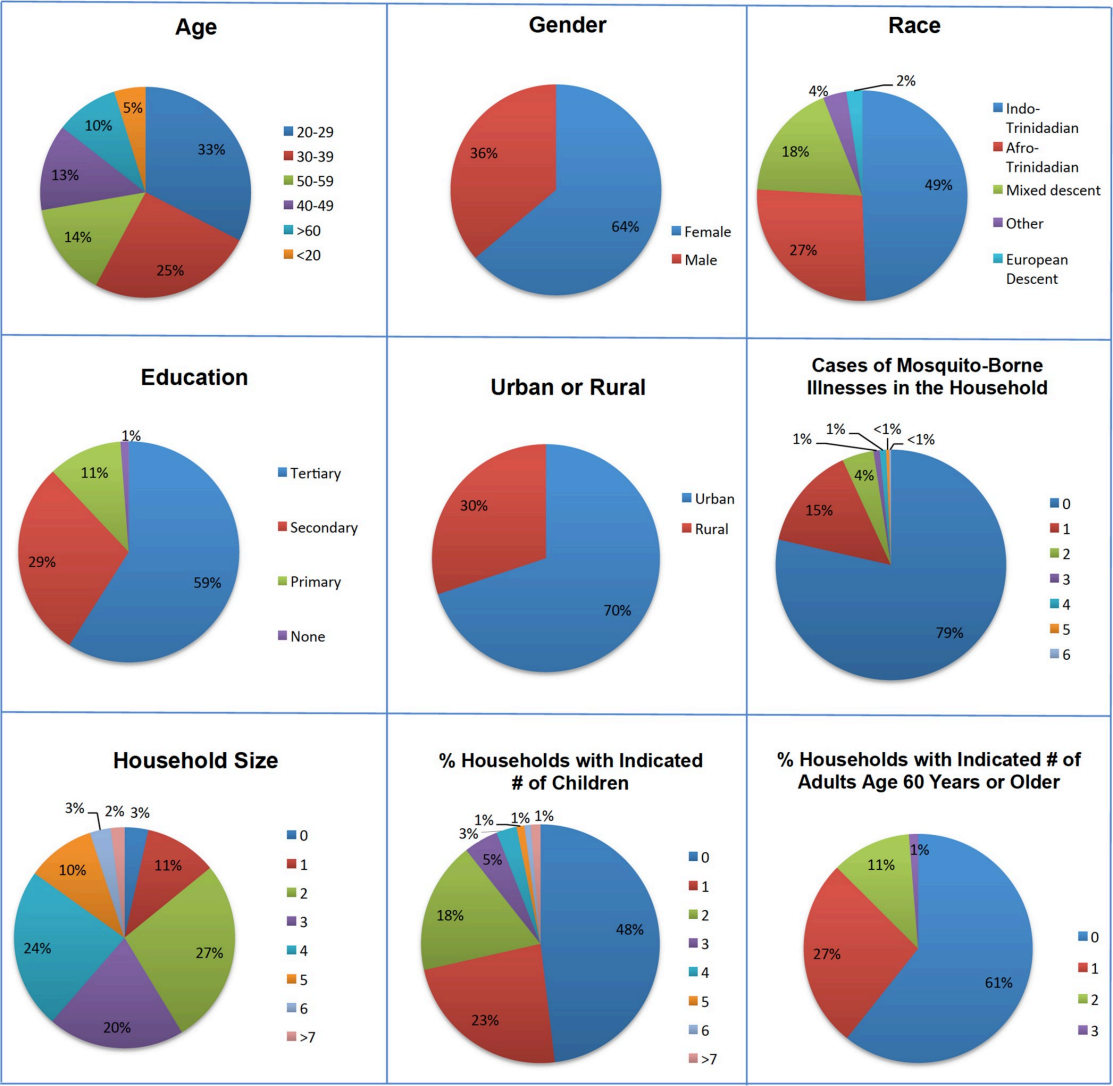
Demographic information for community engagement forum participants. Data regarding the participants’ age, gender, race, education, location of residence (urban or rural), household size, incidences of mosquito-borne illnesses in the household, and presence of children or seniors in the household are provided.

The location, date, and number of attendees at each of the five engagement forums are presented in Table 1. In total, 85 individuals participated in the forums. A summary of the participants’ demographic information is provided in Fig 1. These data indicate that the engagement forum participants consisted of individuals of diverse gender, race, age, and education levels residing in both urban and rural locations. Roughly 25% of the participants lived in a household in which an individual had been afflicted with a mosquito-borne illness in the past two years. ~85% of participants lived in a household with at least one other person. Roughly 50% of these households included children, and ~40% had one or more seniors living in the household.

#### Summary of responses to scripted questions

A summary of the participants’ responses to six scripted questions (S3 File) is provided below. More detailed analyses of these responses follow.

*Question 1*. *What is your impression of how well the larvicides we have developed may work to control mosquitoes on your property*? *What do you think of this approach*? Across the five engagement events, 19 of 85 (22%) total individuals responded at the start of the discussion and indicated that the approach made sense, looked good, or that they would like to try it. Initial comments centered on development of additional formulations (i.e. granules or sprays), formulations that would last for periods of time ranging from two weeks to three months, and means of treating large bodies of water. Several indicated that they felt the approach sounded safe, was organic, and would present fewer health risks with respect to traditional chemical insecticides.

*Question 2*. *Is there anything about the larvicides we described that you particularly like*? Four of 85 (5%) attendees commented that they liked that the product is safe, and four indicated it would be natural/good for the environment. Three individuals indicated they thought the approach would be effective at killing mosquitoes. Three attendees indicated that the yeast would be cost-effective, and one person felt it would be easy to acquire the yeast. One attendee indicated that the product looked easy to use, and another felt that it could be useful for treatment of gutters. Another respondent was glad to see a new approach for mosquito control.

*Question 3*. *Is there anything about the larvicides we described that you did not like*? No one answered this question directly. Instead, the attendees responded with additional questions for the team. Please see below for further discussion about the types of questions asked during the sessions. The attendees were very pleased with the approach, and so that could explain the lack of negative responses. Individuals may also have been too kind to say bad things about the approach. Alternatively, they may have wanted to gather more information before arriving at a final decision.

*Question 4*. *When you think about choosing among product options for mosquito control on your property*, *which factors are most important to you*? Responses to this question are summarized in Table 3. These responses are fairly similar to results obtained in an online economic stakeholders’ survey conducted in Belize [26], in which safety was noted to be a primary concern, as it was in the Trinidad study. Price was the second most common response in the Trinidad engagements. Likewise, the frequency of treatments was discussed, and given the context of the discussions in which it was mentioned, respondents who discussed treatment frequency appeared to be concerned about the relationship between re-treatment needs and costs of the intervention. Concerns for taste and odor were also noted by four attendees. Although environmental concerns were noted at other points in the discussion (see below), only one individual mentioned this as a primary driver of product selection.

**Table 3:** Factors that drive mosquito control product selection.

Response	Count
Safety	7
Price	5
Taste/odor	4
Frequency of treatment	3
Efficacy	2
Environmentally Friendly	1

Counts correspond to the number of participants with the noted response.

*Question 5*. *If the larvicides we described were available for purchase*, *would you be interested in buying them*? *If so*, *what do you think a reasonable price for a monthly supply would be*? Across the engagement events, 28 of 85 (33%) individuals expressed interest in buying the larvicides, and no one indicated that they would not be interested. Seventeen people recommended prices, and $27.82±$6.78 TT ($4.11±$1.00 USD) was the average price recommendation among the respondents (range = $0–100 TT, or $0–14.76 USD). Two individuals recommended that the larvicides should be free; based on the context of this discussion, it appeared that they wanted the government to pay for the larvicides. The MoH does currently apply larvicides in Trinidad, and so this is not an unexpected response.

*Question 6*. *Is there anything else you would like to tell us about the larvicides we are testing*? Several pieces of advice were offered in response to this question. For example, an attendee indicated that it would be good to target other mosquitoes, including malaria vectors, and two other people indicated that they would also like to kill adult mosquitoes. One individual indicated that the development of additional formulations would be worthwhile. Four people were concerned about who would be responsible for treatment of areas adjacent to their own homes, and several indicated that the MoH should be responsible for treating these areas. A full analysis of respondents’ advice and suggestions offered in response to this question, as well as throughout the engagement events is provided below. Moreover, question 6, like question 3, prompted additional questions from the attendees, and a more thorough analysis of these questions is also presented below.

#### Coding of the engagement forum attendees’ comments

Further inspection of the transcripts revealed that the attendees’ comments fell into five broad categories, which were used to code transcript data: 1) information-seeking questions, 2) positive comments, 3) negative comments, 4) neutral comments, and 5) advice regarding product design (Table 4). Quotes representing each of these coding categories are provided in Table 5. Nearly half of the coded comments were positive (Table 4), illustrating the participants’ support of the yeast technology and overall enthusiasm observed across the forums, as illustrated by the representative quotes (Table 5). 7 of 499 (1%) responses indicated that attendants were indifferent, neither in favor of or against the larvicides (neutral, Tables 4 and 5). Although no comments were negative (Table 4), as noted above, attendees asked many questions about the larvicides (Table 5), with 154 of 499 (31%) comments falling under the information gathering code (Table 4). 99 of the 499 (20%) comments included recommendations for product design or improvement (Tables 4 and 5).

**Table 4:** Summary of coding analysis of engagement forum comments.

Code	# of Comments	Total # of Comments	% of Total
**Information gathering**			
UWI-1	23		
UWI-2	28		
Tamana	9	154	31%
Curepe	54		
St. Augustine	40		
**Positive**			
UWI-1	162		
UWI-2	15		
Tamana	7	238	48%
Curepe	29		
St. Augustine	25		
**Neutral**			
UWI-1	1		
UWI-2	0		
Tamana	0	7	1%
Curepe	1		
St. Augustine	5		
**Negative **			
UWI-1	0		
UWI-2	0		
Tamana	0	0	0%
Curepe	0		
St. Augustine	0		
**Recommendations**			
UWI-1	3		
UWI-2	17		
Tamana	19	99	20%
Curepe	17		
St. Augustine	43		
**TOTAL**	498	499	100%

The numbers of comments corresponding to the five codes (Information Gathering, Positive, Neutral, Negative, Recommendations) at each session (UWI-1, UWI-2, Tamana, Curepe, and St. Augustine) are indicated. See text for details.

**Table 5:** Representative quotes from each of the coded categories.

Code	Forum	Representative Quotes
**Information gathering**	UWI-1	So it would be available in the form of a cream or a tablet?
	UWI-1	So is there a specific amount you have to add?
	UWI-2	What about the cost of developing and mass producing?
	Tamana	Is it available already?
	Cuerpe	So would that help with flies as well?
	Curepe	This is the larvae from a whole wide spectrum of mosquitoes or one type of mosquito?
	St. Augustine	How long would this yeast last in a barrel of water?
	St. Augustine	So it don’t do human nothing?
**Positive**	UWI-1	I sometimes work with pesticides and we actually use the pesticides, and they are effective yes. But we also aware they have an ecological arm, and we always like to try new things.
	UWI-2	It doesn’t affect non-target species.
	Tamana	Just the idea then that I may not have to worry about dengue and malaria and all of those things. It would bring comfort then to a home that yes there are going to be mosquitoes, but I can rest assured to some extent that the disease type would not be prevalent in my household.
	Curepe	Might be if you using something as common as yeast, might be very cost effective.
	Curepe	The approach is nice—the approach to keep the environment clean.
	St. Augustine	I would like to use it.
**Neutral**	UWI-1	I have no problem with it.
	St. Augustine	We could try it.
**Recommendations**	UWI-2	I was just going to say that the surface area would be increased in a granular form as opposed to a tablet form.
	Tamana	Like on an individual level to use it in their household but it have to be extended like to the corporations and other things
	Curepe	If you giving it out for free via the ministry we taking it.
	St. Augustine	You could put a little bit so people could sample.
	St. Augustine	I think it should have something to kill all mosquitoes. Because you know, me ain’t gonna look to see which one biting me you know.

Quotes representing each category are shown. Examples from each of the engagement forums are provided.

Further analysis of the information-gathering questions (Table 5) revealed that they tended to fall under several key themes (Table 6). Questions about the process of applying the larvicides were most typical with 13 of 154 (8.4%) of questions falling into this category. For example, attendees might ask how the pellets would be added to the water. They had questions about the types and sizes of the containers and the doses of yeast to be applied. Forum attendees also asked many questions about mosquitoes (10 of 154 questions, 6.5%), for example where and how they lay eggs, how long they live, and the time of day that they seek human blood-meal hosts. Many people asked questions about the safety of the larvicides (9 of 154 questions, 5.8%). For example, an individual might ask if it would be safe to drink treated water and if it would hurt their children, pets, or other animals in the environment. They asked about the safety of the yeast larvicides in comparison to traditional chemical pesticides. Individuals also asked follow-up questions about the yeast, from why it was selected as the delivery method to how it was inactivated (9 of 154 questions, 5.8%). Nine of 154 questions (5.8%) were placed under the “operational” tag. Questions that fell under the operational tag typically corresponded to who would be responsible for applying the larvicides, particularly to larger bodies of land or abandoned properties. Several participants asked for more information about the developmental stage that is being targeted or whether the new pesticides could or should also target adults or additional mosquito species (5 of 154 questions, 3.2%). Several individuals wanted to know more about the research that had been completed (5 of 154 questions, 3.2%) and whether there is potential to develop resistance to the yeast larvicides (4 of 154 questions, 2.6%).

**Table 6:** Common categories of information-gathering questions.

Category	Count	Representative Quote
Application procedure	13	So we need to dissolve it in water and apply it?
Mosquito life history traits	10	Exactly how long an adult go live?
Safety for humans and environment	9	If this is ingested by mistake, by a child for example would it have any effect on the child. Or an animal?
Frequency of treatment	9	So every week you may have to replenish it?
Further information about yeast	9	Basically, like it’s the yeast we cook with right?
Operational	9	The empty piece of land I mean, and like nobody ain't living there. Who going and throw medicine for there?
Residual activity	8	How long would it last? Like a tablet how long would it stay?
Commercialization/commercial product	8	How long did this take to actually reach the market in the different forms that you already spoke about? Like the timeframe?
Container type/size	6	Where would we put it, like where are we using it? Like in the water, in the barrel?
Age of mosquito targeted	5	Does it also affect the adult mosquito?
Prior research on the yeast larvicides	5	What kind of trials?
Dose of treatment	4	So is there a specific amount you have to add?
Water quality post-treatment	4	Would it interfere with the quality of the water in any way?
Price	4	Is it coming at a lower price than everything on the market?
Insecticide resistance	4	What if they develop a resistance to this?
Type of mosquito targeted	4	This is the larvae from a whole wide spectrum of mosquitoes or one type of mosquito?
Formulation	3	So it would be available in the form of cream or a tablet?

The 154 comments coded as information-gathering fell into a number of categories, for which representative questions are shown.

Further analysis of the comments tagged as recommendations (Table 5) revealed several common themes (Table 7). Attendees were most willing to volunteer advice regarding larvicide costs (23 of 99 comments, 23%), perhaps because this was a direct subject covered in the script (question 5). A related piece of advice (6 of 99 comments, 6%) was that it would be good to sell trial sizes so that consumers could evaluate the efficacy of the product before committing to a larger purchase. Once again, advice concerning the operations of larvicide application were abundant (18 of 99 comments, 18%), with much discussion centering on who would be responsible for larvicide application, particularly in large rural or abandoned urban areas. The context of these discussions sometimes deviated to a perceived lack of MoH vector control efforts in the community. Those who were interested in applying the larvicides themselves made recommendations regarding what would facilitate their application procedure and sometimes recommended the development of additional formulations (5 of 99 comments, 5%) or larvicides targeting other mosquitoes (5 of 99 comments, 5%). Safety for humans and the environment (11 of 99, 11%), as well as the prevention of unwanted side effects (5 of 99 comments, 5%) were also common themes.

**Table 7:** Typical categories of advice offered by attendees.

Category of Advice	Count	Representative Quote
Cost/Pricing	23	$20 (TT) a month
Operations	18	You see now, you want me to go and buy a bottle to throw it in that building there? That house across there.
Larvicide application process/procedure	15	I don’t really measure you know. . . I just throw some.
Safety for humans and the environment	11	Well it shouldn't kill me and the mosquito
Larvicide efficacy	6	Quality of the product. . . how well does it you know get rid of the mosquitoes
Utility of offering samples/trial-size packs	6	If there is a trial size so I could get a small amount …so I could buy a small amount at first—see if it works at all?
Additional formulations	5	So your first version is a tablet form but eventually it may go to a spray or so as well you might be able to use your formula in that way.
Interest in targeting other mosquitoes/stages	5	So I guess a customer would still have to deal with the nuisance mosquitoes.
Potential for unwanted side effects	5	So when using this tablet, would it cause water to get cloudy?

Quotes representing each category of advice provided in the engagement forums are noted.

#### Word count analyses reveal common themes

Word count analyses of the combined responses from all five sessions uncovered repeated words and phrases, revealing further patterns in the combined transcript data sets. These analyses, which are presented in Table 8, uncovered six themes: 1) containers, 2) efficacy, 3) environment, 4) formulations, 5) safety, and 6) water. Water, mentioned 66 times (of 6,904 words found in the combined responses from the five sessions), was the most commonly repeated word. Perhaps this is not surprising, as water is vital to both human and mosquito life, the substrate to which the yeast larvicide is applied, and an important commodity on an island, particularly during the dry season when some Trinidadians will rely on the use of water collected and stored during the rainy season. Given that this water is collected and stored in containers, which can become mosquito breeding sites, it is also perhaps not surprising that various container types (barrels, buckets, tanks) were repeatedly mentioned during the engagement discussions (52 of 6,904 words). Words associated with efficacy of the larvicides (45 of 6,904 words) were also abundant, as stakeholders were not surprisingly concerned that the intervention would work. Words associated with formulations (52 of 6,904 words) and safety (18 of 6,904 words), two themes discussed in more detail above, were also commonly mentioned. Finally, words associated with the environment (36 of 6,904 words) were also noted. However, in comparison to an online stakeholder study in Belize, in which the environment was one of the most common concerns of stakeholders [26], environmental themes were less commontly noted in the Trinidad assessments. Eco-tourism is a significant component of the Belize economy, and the Belize survey focused on economic stakeholders in the tourism industry, differences which could help explain these results.

**Table 8:** Summary of word count analyses of the community engagement forum responses.

Theme	Common Words	Count	Quotes Representing this Theme
**1. Containers**	Barrel(s)	24	How effective would that be in a barrel of water?
	Bucket(s)	11	But how much yeast you throw in that bucket?
	Container(s)	7	Where would we put it. . . like where are we using it. . . like in the water, in the barrel?
			What is the size of the pellet and how much of it would you use in like a container and how often would you have to use it?
	Tank(s)	10	How often you would have to use it in the water? Like in your tank or your barrel?
**2. Efficacy**	Work(s)	20	Is there a guarantee if it doesn’t work?
			If it works then we would buy it.
			But in the long term, say 50 years, would it still be effective?
	Last	11	How long would it last? Like a tablet how long would it stay?
			Is there a trial size so I could get a small amount?…So I could buy a small amount at first. See if it works at all.
	Effective	14	If you put this outside and rain fall and the equation changes, how effective will it be?
			I think it’s like 90–100% effective. How wide is that, what area would it cover? Do you have any idea?
**3. Environment**	Environment	7	So if this gets introduced into the environment, so you talked about not targeting your non-targets right. What would be the impacts if this got into the environment?
** **	Flowers/plants	5	
			So you saying that um, the spray is not good for the environment? And this what you developing now is a safer for the environment?
** **	Name of an animal	11	Well I think it is a great idea in the first place because we do have a problem currently with pesticides within the environment within Trinidad and Tobago.
** **	Pollinate/pollinator	3	The, the approach is nice, the approach to keep the environment clean…And the pollinators, we don’t want to kill out the pollinators, right.
**4. Formulations**	Spray(s/ing)	29	So it will be like a spray right?
	Tablet(s)	23	So your first version is a tablet form but eventually it may go to a spray or so as well you might be able to use your formula in that way.
			You would compare it with how much cans of spray you would buy…and if you accustomed buying a spray for a month and you have to buy a supply for a month, you look at the equivalent and see how much.
			Can you get it in a spray form?
**5. Safety**	Baby	3	If I have a barrel of water from which I am going to cook after, would it be harmful to use after?
	Chemical(s)	7	If you could throw it in your tank or if you could drink it still, it harmful to human?
	Harm(ful)	4	So if we could use the tank water to bathe the baby it would make any problem for the baby skin and thing?
	Safe(ty)	4	Because it sounds very safe, I mean, compared to the spray.
			Is it safe for people’s pests, dogs, cats that would drink water from around the house.
**6. Water**	Water	66	Here does have plenty dengue because of the collection of stale water.
			So when using this tablet, would it cause water to get cloudy? Would it interfere with the quality of the water in any way because assuming that you are going to drink this water afterwards?
			So you have a lot of buildup of the water there right. I try and clean close by me but when you look like a little lower down. So things like that cause mosquitoes.
			Because you know you would have it in standing water, you assuming it kills everything is in there.

Word count analyses revealed six themes. Common words associated with each theme, the number of times that the word appeared (among a total of 6,904 words in the community engagement forums), and quotes that exemplify each theme are shown.

### Paper survey analyses

**Fig 2:**
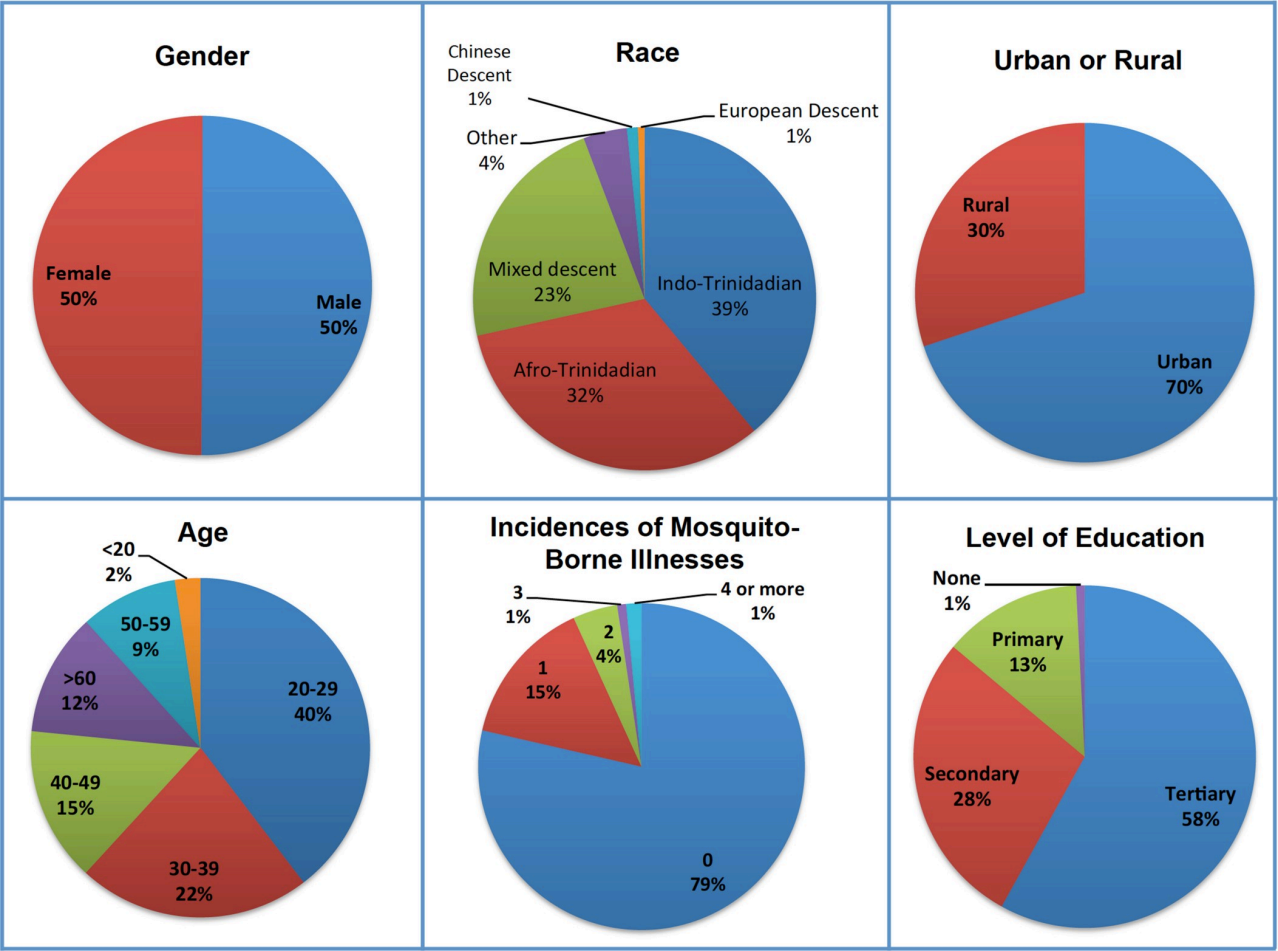
Demographic information for paper survey respondents. Data regarding the gender, race, age, level of education, urban or rural location, and incidences of mosquito-borne illnesses in the households of paper survey respondents are indicated.

509 paper surveys were distributed and collected throughout Trinidad (Table 2). One-third of the surveys were conducted on the UWI campus, which includes students, faculty, and staff residing throughout Trinidad. Assessing people on campus therefore served as a useful means of increasing the reach of the survey. Demographic information for the survey respondents is shown in Fig 2. These demographic data indicate that paper surveys were collected from individuals with diverse gender, race, age, and education levels. A majority of respondents (70%) lived in urban as opposed to rural locations. 21% of respondents reported that at least one incidence of mosquito-borne illnesses had occurred in their households during the past two years.

#### Respondents’ knowledge of mosquitoes and control practices

**Fig 3:**
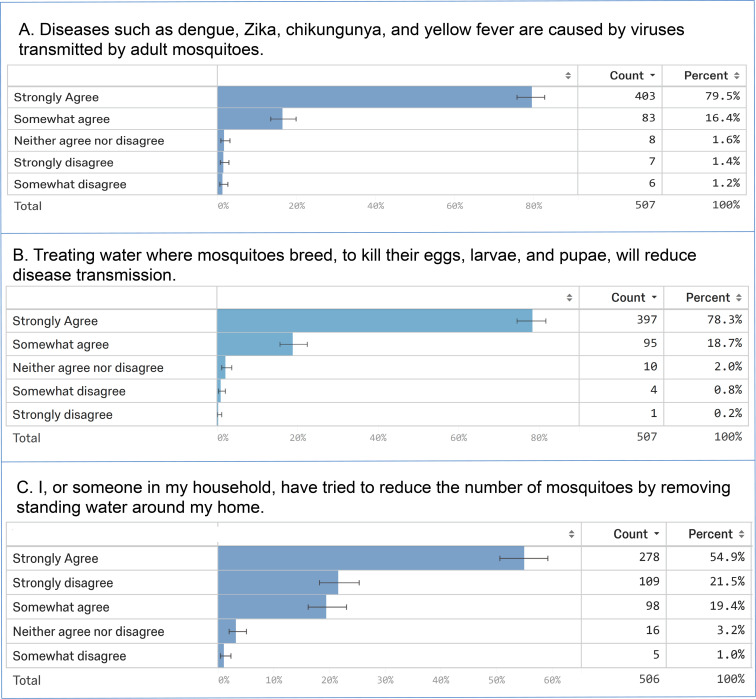
Summary of paper survey respondents’ knowledge of mosquitoes. Responses to three questions regarding mosquitoes and control are indicated. Counts refer to the total number of respondents with the indicated answer, and percentages correspond to the percentage of the total respondents providing the answer.

Respondents were prompted to answer three questions that examined their basic knowledge of mosquitoes and mosquito-borne illnesses. Given the prevalence of mosquito-borne illnesses in Trinidad, the recent Zika outbreak, and based on the results of a recent online survey conducted in Belize [26], it was hypothesized that respondents would have reasonably good knowledge of mosquito biology and disease-causing pathogen transmission. The results were in agreement with this hypothesis, as 96% of the survey respondents agreed that diseases such as Zika, chikungunya, and yellow fever are transmitted by mosquitoes (Fig 3A). Likewise, 97% of respondents agreed that treating water where mosquitoes breed, to kill their eggs, larvae, and pupae, will reduce disease transmission (Fig 3B). 74.3% of respondents indicated that they try to remove standing water around their residences (Fig 3C), indicating that larval source reduction was practiced in a majority of their households. From the 108 of 506 (22%) individuals who indicated that they strongly disagree that they remove standing water on their properties, a significantly higher than expected proportion live in urban areas (100 of 108, 93%, P< 0.00001, Chi-square = 51), are Indo-Trinidadian (54 of 107, 50%, P<0.0000914, Chi-square = 52.7), or have attended tertiary school (51 of 107, 48%, P = 0.00974, Chi Square = 26.3).

#### Use of larvicides

**Fig 4:**
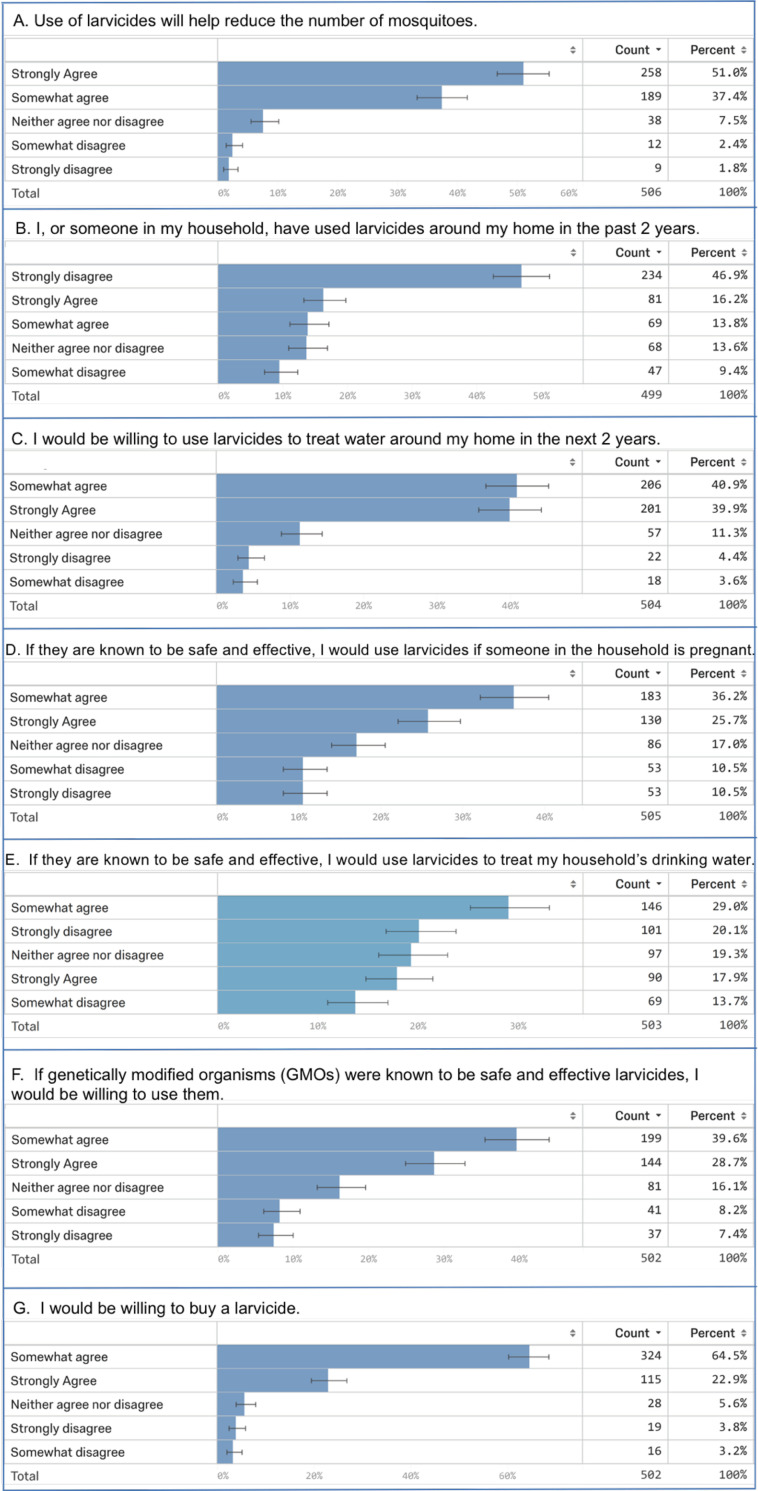
Summary of paper survey respondents’ use of and attitudes toward larvicides. Responses to a series of questions regarding larvicides are shown. Counts refer to the total number of responders with the indicated answer, and percentages correspond to the percentage of the total respondents that provided this answer.

447 of 506 (88%) respondents agreed that larvicides will reduce the number of mosquitoes (Fig 4A). However, only 150 of 499 (30%) respondents agree that they or someone in the household have used larvicides around the home in the past two years (Fig 4B). A possible explanation for this is that the Trinidad and Tobago MoH commonly applies larvicides [11] and individuals may be relying on the MoH to perform this duty on their properties. Despite these findings, 407 of 504 (81%) individuals indicated that they would be willing to use larvicides around their homes (Fig 4C). It is possible that respondents were unhappy with the efforts of the MoH, a common complaint during the engagement sessions, and would be willing to take on the job themselves. In support of this explanation, 439 of 502 (87%) respondents indicated that they would be willing to buy a larvicide (Fig 4G). The average amount that the respondents were willing to spend each month was $59.00±3.27 TT ($8.71±0.48 USD). The range of responses was $0–1500 TT/month ($0.00-$221.33 USD/month; note that the $1500 TT/month response was removed as an outlier from the monthly average).

Recent advances in the use of transgenic release strategies for mosquito control have highlighted the critical importance of effective community engagement prior to the testing and use of new innovations involving genetically modified organisms (GMOs) [22, 27–30]. A majority of respondents indicated that they would use safe and effective larvicides that are genetically modified organisms (343 of 502, 68%, Fig 4F). Likewise, although the yeast was introduced as a genetic system in the introductory presentation, stakeholders did not openly object to use of the yeast larvicides during the community engagement events. These data suggest that the GMO nature of the yeast larvicides may not be a major obstacle in Trinidad. Unlike transgenic mosquito release strategies, which require the release of live GMOs, the yeast interfering RNA larvicides under consideration in our study would be heat-killed prior to use, and no live GMOs would be released into the environment, which may lead to greater acceptance of the intervention. It is of course possible for a vocal minority to have strong opinions against the use of GMOs. Although this was not yet encountered in Trinidad, the small number of paper survey respondents who somewhat disagreed (41 of 502, 8.2%) or strongly disagreed (37 of 502, 7.4%) with GMO larvicide use were analyzed in more detail. A higher than expected proportion of responders living in rural environments (20 of 143, 14.0%) somewhat disagreed with willingness to use GMOs (P = 0.0234, Chi square = 11.3, d.f. = 4). Individuals in the strongly disagree category were significantly more likely to be in the “other” race category (4 of 40, 10.0%, P = 0.000373, Chi-Square = 48.4, d.f. = 20), but their races are unknown. Those who strongly disagreed with using GMO larvicides also were more significantly inclined to strongly disagree with using a larvicide in the next two years (9 of 36, 25%, P< 0.00001, Chi square = 164, d.f. = 16) or to have reduced standing water on their properties (3 of 37, 8%, P = 0.000735, Chi square = 40, d.f. = 16). Thus, these individuals may be more opposed to water treatment or larval source reduction in general than the use of GMO larvicides in particular. A recent stakeholder study in Belize uncovered similar findings [26].

Responses to whether safe and effective larvicides would be used to treat drinking water were more varied (Fig 4E). 236 of 503 (47%) respondents agreed that they would use larvicides in drinking water, but 170 of 503 (34%) disagreed, and 97 of 503 (15%) fell in the neither agree nor disagree category. One potential explanation for this is that some individuals store their water in covered tanks that are not traditionally treated by the MoH [11], and so they may not perceive a need for this. Also, given the consistent mention of safety in the community engagement events (Tables 3 and 6–8), individuals may also be more apprehensive about treating their drinking water with pesticides. These data were further analyzed to determine if any trends could be revealed. However, outside of the “other” race group, in which a higher than expected number of individuals were significantly inclined to strongly disagree with drinking water treatment (9 of 97, 9%, P = 0.01, Chi square = 35.8, d.f. = 20), there were no other significant trends observed in the demographics of these individuals.

Finally, the paper surveys provided more insight into the target users and buyers of larvicides. These results, including relevant respondent counts and statistical values, are presented in Table 9. A significantly higher than expected proportion of individuals who indicated they are strongly likely to larvicide in the next two years were >60 years old, and significantly fewer than expected individuals in this category were 20–29 years of age. Similarly, a significantly higher than expected proportion of those >60 years of age strongly agreed that they had used larvicides in the past two years (20 of 56, 26%, P = 0.00006, Chi square = 53.9, d.f. = 20). This may reflect a greater likelihood for those who have retired to spend a greater amount of time at home or to be in charge of the household, in which cases they may be more willing to use larvicides at their homes than younger individuals. Those who strongly agree with larvicide use also strongly agree that mosquitoes transmit viruses that cause disease and that treating water reduced disease transmission. Likewise, a significantly higher than expected proportion of those strongly inclined to use larvicides strongly agree that attempts have been made to reduce standing water on their properties. Significantly higher than expected proportions of these individuals strongly agree that larvicides were used on their properties in the past two years and strongly agree that the use of larvicides reduces the number of mosquitoes. Finally, these individuals are significantly more likely to strongly agree that they would use larvicides if a pregnant person resides in the household, to treat drinking water, and if the larvicide is a GMO. In summary, it appears that individuals who are willing to use larvicides believe that mosquitoes transmit disease and take efforts to reduce mosquitoes through removal of standing water on their properties. These individuals believe that larvicides work and are willing to use safe and effective larvicides to treat drinking water, including cases in which pregnant women reside in their homes. They do not appear to have significant concerns about the use of GMO larvicides.

**Table 9:** Profile of respondents who are strongly likely to use larvicides in the next two years.

Variable	Count	%	Chi Square	P value
Strongly agree that mosquitoes transmit viruses that cause disease	172	86	40	0.000822
Strongly agree that treating water reduces disease transmission	180	90	90	< 0.00001
Strongly agree that attempts were made to remove standing water	141	71	71	< 0.00001
Strongly agree that using larvicides reduces the number of mosquitoes	153	77	132	< 0.00001
Strongly agree with having used larvicides in the past two years	69	35	120	< 0.00001
Strongly likely to use larvicides with a pregnant person in the household	91	46	215	< 0.00001
Strongly agree with treatment of drinking water	66	33	147	< 0.00001
Strongly likely to use a GMO larvicide	99	51	164	< 0.00001
>60 years	33	17	37	0.0109

Significant two variable comparisons are shown. Counts refer to the number of individuals from a total of 200 respondents who are strongly likely to use larvicides, and % corresponds to the percentage of these 200 respondents. These data were analyzed using Chi Square analysis (Chi Square and P values are indicated, degrees of freedom = 16).

Those who indicated that they were strongly likely to buy larvicides were also analyzed in more detail. These results, including respondent counts and all relevant statistical values, are presented in Table 10. The characteristics of respondents in this category were similar to those who strongly agree with the use of larvicides in the next two years (Table 9). A significantly higher than expected proportion of individuals who indicated they are strongly likely to buy a larvicide in the next two years strongly agree that mosquitoes transmit viruses that cause disease and that treating water reduces disease transmission. Higher than expected proportions of these individuals have used larvicides in the past two years, and they expect to do so in the next two years. Moreover, as with those who are likely to use larvicides, higher than expected proportions of those significantly willing to buy larvicides strongly agree to use larvicides if a pregnant person resides in the household, to treat drinking water, and if the larvicide is a GMO. One interesting difference between those highly willing to use larvicides (Table 9) and highly willing to buy larvicides (Table 10) was observed. Unlike prospective larvicide users, those who are highly likely to buy larvicides are highly likely to disagree that they have made attempts to remove standing water on their properties. Thus, those willing to buy the larvicides appear to be more concerned with water treatment than the removal of standing water. Interestingly, those significantly willing to buy larvicides lived in urban environments (Table 10). The availability of piped water in Trinidad can be unpredictable, even in urban areas, and so treatment of stored water may be desirable.

**Table 10:** Profile of respondents who are strongly likely to buy larvicides.

Variable	Count	%	Chi Square	P value
Strongly agree that mosquitoes transmit viruses that cause disease	102	89	42	0.000388
Strongly agree that treating water reduces disease transmission	109	95	95	< 0.00001
Strongly disagree that attempts were made to remove standing water	51	44	71	< 0.00001
Strongly agree that using larvicides reduces the number of mosquitoes	84	73	63	< 0.00001
Strongly disagree with having used larvicides in the past two years	3	23	45	0.000128
Strongly agree they will use larvicides in the next two years	79	69	179	<0.00001
Strongly agree with treatment of drinking water	35	30	121	<0.00001
Strongly likely to use a GMO larvicide	61	53	238	< 0.00001
Urban	96	85	19	0.000981
40–49 years of age	26	23	46	0.000857

Significant two variable comparisons are shown. Counts refer to the number of individuals from a total of 115 respondents who are strongly likely to buy larvicides, and % corresponds to the percentage of these 115 respondents. These data were analyzed using Chi Square analysis (Chi Square and P values are indicated, degrees of freedom = 16).

Data on prospective users (Table 9) and buyers (Table 10) could inform the design of educational campaigns to broaden the prospective yeast larvicide user group. These data indicate that it may be useful to direct these campaigns toward individuals under age 40 (see discussion above), and so it is good that students at the UWI campus were engaged. Given that several survey questions were based on the assumption that the larvicides have been proven to be safe and effective, it will be critical to demonstrate the safety and efficacy of the yeast larvicides, two themes highlighted in the engagement forums (see above). If these criteria are met, the paper survey data indicate that prospective users and buyers exist in Trinidad.

## Conclusions

In conclusion, the community engagement events and paper surveys conducted in this investigation proved to be useful tools for assessing stakeholders in Trinidad and Tobago. These assessment tools facilitated evaluation of participants’ general knowledge of mosquitoes, mosquito control practices, feelings about larviciding, and larvicide technology. The results of the investigation demonstrated that the participants were generally well-informed regarding mosquito biology, control, and larviciding (Figs 3 and 4) and were eager to learn more (Tables 4 and 5). Trinidadian participants were receptive to the yeast larvicides (Tables 4 and 5) and to larviciding in general (Fig 4). A majority of Trinidadians were willing to buy a larvicide (Fig 4), and profiles of prospective larvicide users (Table 9) and buyers (Table 10) indicated that these individuals tend to believe that mosquitoes spread disease and that mosquito control interventions can reduce the number of mosquitoes and disease burden. Safety, cost, and efficacy (Tables 3, 7 and 8) of the larvicides are critical and are therefore of significant importance to ongoing efforts to commercialize yeast interfering RNA larvicide technology. In general, these analyses suggest that the residents of Trinidad are prospective users (Tables 4 and 9, Fig 4) and buyers (Tables 4 and 10, Fig 4) of yeast larvicides. Trinidadians were curious about this technology and used the engagement events to learn more about it (Tables 4–6). Although these larvicides could potentially one day be used to treat drinking water, not all Trinidadians envision using the larvicides in this manner (Fig 4), most likely because users will need to be ensured that this new technology is safe (Tables 3 and 6–8). These findings suggest that it will be important to further consider the market for larvicides used to treat drinking water.

The results of this investigation, from both the engagement forums and paper surveys, indicate that Trinidadians are not particularly concerned about GMO use (Fig 4, Tables 9 and 10) as long as the larvicides are shown to be safe and effective, but it will be important to continue to assess perceptions of genetically modified yeast in Trinidad and Tobago, as well as other parts of the world. As noted by Oliva et al. [28], the technical manner in which new technology is explained can impact stakeholder responses, and the level of technical detail provided to stakeholders is a matter of debate within the vector research community. In our paper survey, which gathered only baseline data, technical details were not explained, and participants did not have an opportunity to ask questions. However, in the community engagement forums, which were geared toward education of stakeholders and achieving an open dialogue about the new intervention, the introductory explanation of the technology (S3 File) was delivered in relatively simple terms that were designed to be understood by audiences with differing backgrounds. These introductory remarks were prepared with input from scientists who developed the yeast, scientists with knowledge of vector control technologies, ethicists, local Trinidad and Tobago researchers, in consultation with vector control staff at the Trinidad and Tobago MoH, and based on feedback from local citizens of Trinidad and Tobago who lacked advanced scientific training. The discussions that evolved during the forums were designed to be open-ended and became as technical as the audiences, their questions, and comments required. Any and all technical questions were answered by members of the research team who had extensive knowledge of the technology. The investigative team was generally pleased with the levels of discourse achieved in these engagements and has adopted a similar approach in other ongoing studies. It was noted by several engagement forum participants that props, such as mosquito eggs and yeast larvicide samples, would be beneficial, and inclusion of such props has proven useful in subsequent investigations.

If large-scale yeast interfering RNA larvicide field trials are initiated in Trinidad and Tobago or elsewhere, it will be critical to continue to engage with the stakeholders, to continue to answer their questions about the technology, and to gather feedback regarding the technology once it is actually being used at their places of residence. Moreover, given that both the paper surveys and community engagement forum questions centered on the notion of how the participants felt about using the technology “if it were shown to be safe and effective,” it is of course vital that safety and efficacy are demonstrated prior to expanded testing, commercialization, and widespread use of this technology. To this end, it will be important to pursue U.S. Environmental Protection Agency (EPA) and World Health Organization registry approvals for this new intervention. Introductory conversations with the EPA regarding RNAi-based yeast larvicides have been initiated, and these conversations will continue as commercial-ready yeast formulations are developed and testing of these formulations ensues. The EPA has already approved RNAi-based technology for control of an insect pest through genetic modification of a crop plant [30]. During EPA deliberations, it was noted that organisms routinely consume interfering RNA molecules, which are endogenously produced in many plant and animal species, suggesting that consumption of interfering RNA that lacks specific target sites in non-target species would pose little if any risk to these organisms [31]. Our own initial toxicity assays of several yeast interfering RNA larvicides in non-target organisms [19–21] support this concept. In the EPA deliberations [31], it was also noted that in humans, orally consumed interfering RNA is not expected to last in the gastrointestinal tract. Nevertheless, it was noted that additional research on RNAi safety is warranted [31], and further research on RNAi safety in general, along with detailed toxicological analysis of our own larvicides in support of applications for regulatory approvals will be the subjects of future investigations. As further research is pursued, the disclosure of any noted risks to stakeholders in Trinidad and Tobago or at other sites where large-scale field tests are conducted would be essential.

As described by Lavery et al. [22], effective community engagement in global health studies involves early initiation of engagement activities. Such activities serve to: i) provide investigators an opportunity to ensure that the purpose and goals of the research are clear to the community, ii) establish relationships and commitments to build trust with relevant community authorities, iii) allow researchers to understand the community, its diversity, changing needs, and assets, iv) maximize opportunities for stewardship, ownership, and shared control by the community, v) provide a platform for expression of dissenting opinions or in extreme cases, prohibition of the research, and vi) give the researchers an opportunity to modify the proposed research strategies, as needed. This investigation has begun the ongoing process of achieving these goals. In particular, the community engagement events served to educate residents about mosquitoes, mosquito control, and yeast larvicide technology (Tables 4 and 5). The paper surveys were useful for gathering baseline data on a variety of topics related to the yeast larvicide intervention from a large number of people, which is also useful, but were not educational or interactive. Nevertheless, the paper surveys would have captured widespread opposition to the technology in question, but none was encountered. If it had been, any such opposition could have been further explored during the community engagement forums, which provided a framework for open dialogue, education, and stakeholder input into the development process. On a more personal note, these engagement events were highly rewarding to the team. The engagement forums helped us better understand the needs of stakeholders in Trinidad, and the question and answer sessions served as a somewhat unanticipated outreach opportunity for this research program. Overall, participants voiced a very enthusiastic response to the yeast larvicide technology (Tables 4 and 5), and no significant opposition was encountered in either the paper survey or engagement forum studies. As such, and given that other suggested minimal criteria for selection of field sites as defined by Brown et al. [29] have been met, including the presences of the target species, a credible regulatory structure which has approved field testing of this intervention, resource commitments, and local research teams with expertise in vector biology, we believe that any of the locations where the events were held represent viable field study sites.

If large-scale field testing of the larvicide initiates, it will be critical to continue to engage with the stakeholders. To achieve this, our future research plans include individual interviews with household members who agree to test the larvicides on their properties. These interviews represent the next phase of stakeholder engagements and expand upon the paper surveys and engagement forums. This phased engagement approach coincides with a phased larvicide testing program that initiates with semi-field contained studies on rooftop laboratories and progresses into small-scale and large-scale field evaluations. Such phased approaches have been recommended by other developers of new vector control technologies [29], and based on the findings of the present investigation, we agree that such an approach is both important and useful. Moreover, individual interviews may provide a valuable means of interacting with individuals who do not feel comfortable speaking out in public forums, but who may have valuable insights to share. Such interviews are also likely to serve as an effective means of engaging with elderly stakeholders who are retired, and therefore more likely to be home during the day when MoH workers and members of the research team conduct field trials.

*Aedes* mosquitoes continue to evade conventional interventions for vector control, and it is critical that new vector control interventions are established. Given the increased incidences of mosquito-borne illnesses, the establishment of benchmarks for acceptable community engagement and acceptance by public health organizations is critical and must be balanced with an ever-growing need to address the health and economic costs of these diseases [27, 30]. Little data exists on the extent and type of outreach required for sufficient stakeholder engagement prior to and during the testing of new vector control interventions [30]. As such, the results of this investigation will contribute to critical ongoing discussions concerning what benchmarks for acceptable engagement and support by communities and stakeholders are sufficient [29, 30], as well as how best to achieve an adequate understanding of stakeholders’ perceptions and acceptance of emerging technologies for mosquito control.

## Supporting information

S1 File Larvicide community engagement forum study information sheet. Engagement forum participants were provided a copy of this information sheet prior to participating in the forum.
(PDF)

S2 File Demographic questions for community engagement participants. Engagement forum participants were encouraged, though not required, to complete this demographic information sheet. (PDF)

S3 File Larvicide community engagement forum script. Moderators of the community engagement forums used this script while conducting each forum. (PDF)

S4 File Mosquito larvicide survey study information sheet. Paper survey responders were provided a copy of this information sheet prior to completing the survey. (PDF)

S5 File Mosquito larvicide survey. This survey, which includes an optional participant demographics section, was distributed on paper.
(PDF)
