# Supplementary material for: Community acceptance of yeast interfering RNA larvicide technology for control of Aedes mosquitoes in Trinidad
Source: PLoS One. 2020 Aug 14;15(8):e0237675. doi: 10.1371/journal.pone.0237675 (PMC7428178; doi:10.1371/journal.pone.0237675)
Supplement: S2 File — (PDF) [file pone.0237675.s002.pdf]

Demographic Questions for Community Engagement Forum Participants

Please tell us more about you (optional):

**Question**	**Answer**					
What is your gender?	*Male*			*Female*		
What is your age in years?	*<20*	*20-29*	*30-39*	*40-49*	*50-59*	*>60*
Indicate the highest level of formal education you have completed or in which you are presently enrolled:	*Tertiary*	*Secondary*	*Primary*	*None*		
What is your race?	*Afro-Trinidadian*			*Chinese Descent*		
	Indo-Trinidadian			*Mixed Descent*		
	European Descent			*Other*		
