# Supplementary material for: Community acceptance of yeast interfering RNA larvicide technology for control of Aedes mosquitoes in Trinidad
Source: PLoS One. 2020 Aug 14;15(8):e0237675. doi: 10.1371/journal.pone.0237675 (PMC7428178; doi:10.1371/journal.pone.0237675)
Supplement: S3 File — (PDF) [file pone.0237675.s003.pdf]

Larvicide Community Engagement Forum Study

Introduction

Welcome and thank you for accommodating us today. Do you know that there are 178 mosquito species in Trinidad and Tobago? How do you all deal with these pests? The most common methods presently used are pesticides, sprays, and smoke. Aedes aegypti is a mosquito that spreads diseases, including Zika, dengue, chikungunya, and yellow fever, in Trinidad and Tobago. With no commercially available medications or vaccines for most of these diseases, targeting the vector is the primary means of disease prevention. This includes use of either pesticides, biological agents and exclusion methods such as nets, targeting either the adult or larval stages. Most of the existing methods for vector control rely on the use of chemical pesticides. Sadly, resistance to these insecticides has begun to emerge, and there are rising concerns about the safety and toxicity of these pesticides for humans. There are also concerns for unintended effects of pesticides on non-target species like bees and other pollinators which are important for many reasons, including the agricultural sector and environmental biodiversity.

Larviciding, which typically involves the addition of chemical insecticides to water in which mosquitoes breed, is critical for controlling Aedes mosquitoes. Due to emerging pesticide resistance and concerns for the impacts of broad-spectrum chemical pesticides on non-target species, the current collection of larvicides is not sufficient for prevention of mosquito-borne illnesses. We are developing a new strategy that involves the use of RNA interference (RNAi) for control of Aedes aegypti. This involves the use of RNA, which is similar to the DNA found in genes. It is important in the making of proteins in cells. Therefore, if we "interfere" with the building blocks of mosquito RNA; proteins cannot be properly made and the larvae die. The RNA used in these larvicides is designed to match mosquito genes, not the genes of humans or other non-target organisms. This means that this novel larvicide is designed to kill only mosquitoes and nothing else. We engineered baker's yeast to make RNA – a part of the cell of the mosquito. The RNA is produced as the yeast is grown. This allows for simple and cost-effective RNA larvicide production.

Chemical pesticides are currently used to treat water-filled containers where mosquitoes breed. Yeast larvicide tablets, which resemble yeast nutritional tablets, could replace these chemical pesticides. We believe that these yeast tablets represent a safe and effective way to overcome pesticide resistance and concerns for the use of chemicals on the environment. Our engineered yeast was inactivated and dried into ready-to-use pellets. The yeast pellets were fed to larvae in laboratory trials. In the lab, one small 50 mg tablet of inactivated yeast was found to kill up to 100% of larvae. We have developed many different promising yeast larvicide strains targeting different mosquito genes. We now plan to test these in controlled field trials.

Larvicide Community Engagement Forum Study

Script

Interviewer: *We want to thank you very much for participating in our study of new ways to control mosquitos in standing water. As we move forward, it is very important for us to hear directly from you about your thoughts and feelings pertaining to the larvicides we are studying.*

1. *The purpose of a larvicide is to prevent mosquito larvae from surviving and developing into adult mosquitoes which can bite and carry disease.* **What is your impression of how well the larvicides we have developed may work to control mosquitoes on your property? What do you think of this approach?**

2. **Is there anything about the larvicides we described that you particularly like?** *We are interested in learning about ways our approach to larviciding might appeal to users more than alternative approaches.*

3. **Is there anything about the larvicides we described that you did not like?** *We are interested in learning about ways to improve our larvicides.*

4. **When you think about choosing among product options for mosquito control on your property, which factors are most important to you?**

5. **It the larvicides we described were available for purchase, would you be interested in buying them? If so, what do you think a reasonable price for a monthly supply would be?**

6. **Is there anything else you would like to tell us about the larvicides we are testing?**

Interviewer: *We thank you for participating in this interview. Your input is very important to us.*

Larvicide Community Engagement Forum Study

Follow Up Question Bank

______ = word or phrase the respondent has already said in the initial answer to an interview question OR word or phrase in the interview question

Could you tell me more about ______?

What do you mean by ______?

Could you explain ______ to me further?

Could you give me some more details about ______?

What other words would you use to describe ______?

Can you give me an example of ______?

What happened after ______?

Interviewer: *We thank you for participating in this interview. Your input is very important to us.*
