# Supplementary material for: Community acceptance of yeast interfering RNA larvicide technology for control of Aedes mosquitoes in Trinidad
Source: PLoS One. 2020 Aug 14;15(8):e0237675. doi: 10.1371/journal.pone.0237675 (PMC7428178; doi:10.1371/journal.pone.0237675)
Supplement: S4 File — (PDF) [file pone.0237675.s004.pdf]

Study Information Sheet

Mosquito Larvicide Survey

You are invited to participate in a survey research study about the use of mosquito larvicides in Trinidad. The people of Trinidad can contract serious diseases through the bites of mosquitoes, and a research project being conducted here will investigate new ways of preventing mosquitoes from transmitting infections.

This study is being conducted by Dr. Azad Mohammed (The University of the West Indies at St. Augustine, Trinidad and Tobago), as part of a larger research project led by Dr. Molly Duman Scheel of Indiana University School of Medicine-South Bend, in collaboration with Dr. David W. Severson and Nicole Achee of the University of Notre Dame (United States). You are invited to participate in this study because you are an adult resident of Trinidad. If you have any questions about this study, please contact Dr. Molly Duman Scheel at (574) 631-7194 (country code 1) or mscheel@nd.edu. For questions about your rights as a research participant, to discuss problems, complaints, or concerns about a research study, or to obtain information or offer input, contact the IU Human Subjects Office at 317-278-3458.

This study consists of a brief survey, with specific questions which will allow us to learn more about thoughts of adult Trinidad residents regarding household use of mosquito larvicides. As the larger research project pursues new ways of preventing mosquitoes from carrying disease, it is very important for us to consult adults in households where larvicides may be used. The purpose of the study is to collect information about the thoughts, feelings, and experiences of adult residents of Belize regarding household use of mosquito larvicides.

Participation in this study is voluntary and you may choose to stop participating in this study at any time. This study consists of a brief survey, which should take 2-8 minutes to complete.

If you agree to participate in this study, you will be asked to indicate your degree of disagreement or agreement, on a scale of 1 to 5, with statements pertaining to household us of mosquito larvicides.

Participating in this study may not provide any direct benefit to you. The knowledge gained from this study will be used as part of a large research project that may result in the development of new mosquito larvicides, ultimately intended to disrupt the transmission of human disease. We do not envision any significant risks related to participation in this study.

Privacy with respect to information you will share with us if you participate in this study will be protected by the investigators. Your name and other information which would allow you to be identified as an individual are not being collected in this study, so your responses will not be attributable to you.

Thank you for agreeing to participate in our research. Before you begin, please note that this research is for residents of Trinidad over the age of 18; if you are not a resident of Trinidad and/or under the age of 18, please do not complete this survey.
