# Supplementary material for: Community acceptance of yeast interfering RNA larvicide technology for control of Aedes mosquitoes in Trinidad
Source: PLoS One. 2020 Aug 14;15(8):e0237675. doi: 10.1371/journal.pone.0237675 (PMC7428178; doi:10.1371/journal.pone.0237675)
Supplement: S5 File — This survey, which includes an optional participant demographics section, was distributed on paper. (PDF) [file pone.0237675.s005.pdf]

Mosquito Larvicide Survey

Please mark the circle which most closely represents your agreement or disagreement with the following statements about mosquito larvicides, pesticides that kill mosquito larvae.

	STRONGLY AGREE	***SOMEWHAT AGREE***	***NEITHER AGREE NOR DISAGREE***	***SOMEWHAT DISAGREE***	***STRONGLY DISAGREE***
Diseases such as dengue, Zika, chikungunya, and yellow fever are caused by viruses transmitted by adult mosquitoes.	○	○	○	○	○
Treating water where mosquitoes breed, to kill their eggs, larvae, and pupae, will reduce disease transmission.	○	○	○	○	○
I, or someone in my household, have tried to reduce the number of mosquitoes by removing standing water around my home.	○	○	○	○	○
Use of larvicides will help reduce the number of mosquitoes.	○	○	○	○	○
I, or someone in my household, have used larvicides around my home in the past 2 years.	○	○	○	○	○
I would be willing to use larvicides to treat water around my home in the next 2 years.	○	○	○	○	○
If they are known to be safe and effective, I would use larvicides if someone in the household is pregnant.	○	○	○	○	○
If they are known to be safe and effective, I would use larvicides to treat my household's drinking water.	○	○	○	○	○
If genetically modified organisms (GMOs) were known to be safe and effective larvicides, I would be willing to use them.	○	○	○	○	○
I would be willing to buy a larvicide.<br>• (If you strongly or somewhat agree:) An affordable amount for our household to spend on larvicides each month is: $______	○	○	○	○	○

<u>Optional: Please tell us more about your household</u>:

In the past two years, how many times has someone in your household had dengue, Zika, chikungunya, or Yellow fever? If you cannot remember exactly, it is alright to estimate. _____

How many children under the age of 18 years live in your household? _____

How many adults 18-59 years of age live in your household? _____

How many adults 60 years of age or older live in your household? _____

<u>Optional: Please tell us more about you:</u>

What is your gender? *Male* *Female*

What is your age in years? *<20* *20-29* *30-39* *40-49* *50-59* *>60*

What is your race? *Afro-Trinidadian* *Indo-Trinidadian* *European Descent* *Chinese descent* *Mixed descent* *Other*

Where do you currently live? *Urban* *Rural*

Indicate the highest level of formal education you completed or in which you are presently enrolled: *Tertiary* *Secondary* *Primary* *None*
